# The female menstrual cycle does not influence testosterone concentrations in male partners

**DOI:** 10.1186/1477-5751-11-1

**Published:** 2012-01-03

**Authors:** Jakob O Strom, Edvin Ingberg, Emma Druvefors, Annette Theodorsson, Elvar Theodorsson

**Affiliations:** 1 Clinical Chemistry, Department of Clinical and Experimental Medicine, Faculty of Health Sciences, Linköping University, Department of Clinical Chemistry, County Council of Östergötland, SE-58185, Linköping, Sweden; 2 Department of Surgery, Ryhov County Hospital, County Council of Jönköping, SE- 551 11, Jönköping, Sweden; 3 Neurosurgery, Department of Clinical and Experimental Medicine, Faculty of Health Sciences, Linköping University, Department of Neurosurgery, County Council of Östergötland, SE-58185, Linköping, Sweden

**Keywords:** Testosterone, Menstrual cycle, Ovulation, Salivary, Pheromones, Hormones

## Abstract

**Background:**

The time of ovulation has since long been believed to be concealed to male heterosexual partners. Recent studies have, however, called for revision of this notion. For example, male testosterone concentrations have been shown to increase in response to olfactory ovulation cues, which could be biologically relevant by increasing sexual drive and aggressiveness. However, this phenomenon has not previously been investigated in real-life human settings. We therefore thought it of interest to test the hypothesis that males' salivary testosterone concentrations are influenced by phases of their female partners' menstrual cycle; expecting a testosterone peak at ovulation.

**Methods:**

Thirty young, healthy, heterosexual couples were recruited. During the course of 30-40 days, the women registered menses and ovulation, while the men registered sexual activity, physical exercise, alcohol intake and illness (confounders), and obtained daily saliva samples for testosterone measurements. All data, including the registered confounders, were subjected to multiple regression analysis.

**Results:**

In contrast to the hypothesis, the ovulation did not affect the testosterone levels, and the resulting testosterone profile during the menstrual cycle was on the average flat. The specific main hypothesis, that male testosterone levels on the day of ovulation would be higher than day 4 of the cycle, was clearly contradicted by a type II error(β)-analysis (< 14.3% difference in normalized testosterone concentration; β = 0.05).

**Conclusions:**

Even though an ovulation-related salivary testosterone peak was observed in individual cases, no significant effect was found on a group level.

## Background

Testosterone is by far the most potent naturally occurring androgen [1], and has over the years attracted considerable research efforts, e.g. for its behavioral and developmental effects [2] and its implications in aging [3,4]. The concentrations of testosterone are influenced by several factors, including endogenous cycling patterns such as a circadian cyclicity, rendering peak levels in the morning and nadir levels during the night [1], and the circannual variation with a peak in summer and a valley in winter/early spring [5,6]. Other infradian testosterone cycles between 6 and 33 days of lengths have also been proposed, as yet with limited support in scientific studies [7-12].

A number of exogenous factors, such as lacrimal secretions, can influence the male testosterone concentrations [13], possibly reflecting pheromonal effects. It has further been demonstrated that sniffing a t-shirt that had been worn by an ovulating female caused a smaller reduction in testosterone levels than did smelling a t-shirt from a non-ovulating woman, suggesting that menstrual cycle-related factors affect the male's testosterone concentrations in experimental settings [14]. Moreover, several animal studies have shown that the male testosterone concentrations are affected by the female hormone cycle, for example that stumptailed macaques exhibited elevated testosterone concentrations in response to follicular phase secretions [15]. It is also generally accepted that women living together synchronize their menstrual cycles [16,17]. Taken together, the existing literature implies that male testosterone concentrations could be influenced by the female menstrual cycle, but to the best of our knowledge, this hypothesis has hitherto not been directly tested in real-life human settings.

**Figure 1:**
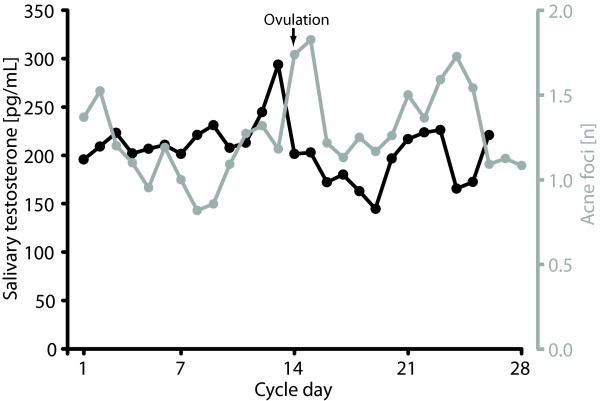
**Pilot study results**. In a small pilot study, the effect of one female's menstrual cycle on the male partner's mean number of acne foci during 18 months (gray line) and mean salivary testosterone concentrations (black line) during two other months was assessed. A clear peak is seen day 13-15 (around ovulation) with number of acne foci lagging approximately 2 days after the salivary testosterone concentration peak.

Two pilot studies were performed prior to the main experiment. In the first, one person's daily fluctuations in facial acne (biomarker of androgenic activity [18]) was registered over a period of 18 months, and in the second, salivary testosterone was collected from the same male during two other months, and the data were compared to the partner's menstrual cycle. Both acne and salivary testosterone concentrations implicated a two or three-peak monthly pattern, with peaks on ovulation day, ten days after ovulation and a possible peak during menses (Figure 1). Since the pilot studies were based on merely one couple, they were purely used for generating hypothesis when designing the main study, and should not be viewed as parts of the result of the same.

The hypothesis tested in the present work was that male salivary testosterone concentrations are affected by the respective female's menstrual cycle, so that two peaks are produced over the month; the first peak was expected during ovulation (main hypothesis) and the second peak in the mid-luteal phase (secondary hypothesis). This was tested by daily saliva testosterone samples from 30 young, healthy males, with concurrent registration of partner menstrual cycle data and control for confounders (masturbation, intercourse, physical exercise, alcohol consumption, illness and absence from partner overnight).

## Results

### Testosterone Concentrations

**Figure 2:**
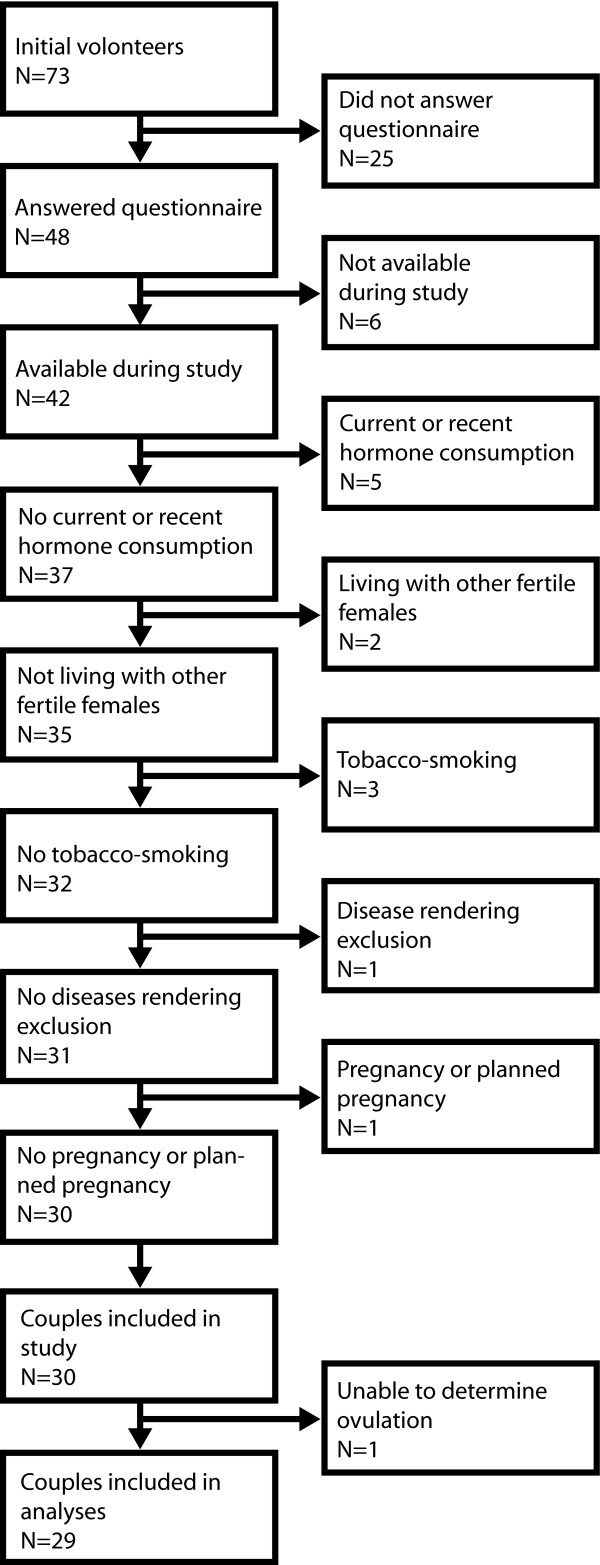
**Inclusion/exclusion flow chart**. Flow chart of the number of initially volunteering couples and the inclusion/exclusion process. Out of the 48 couples that answered the questionnaire, 30 were finally included in the study. One couple were excluded after data collection because of difficulties in determining the ovulation date, leaving 29 couples to be included in the analyses.

**Figure 3:**
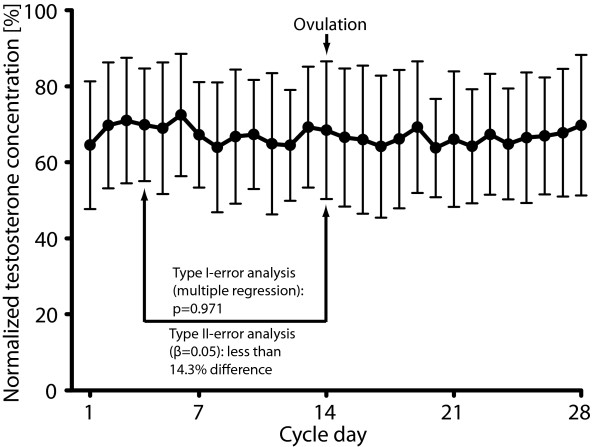
**No effects of ovulation on testosterone concentrations**. Normalized testosterone concentrations in the saliva from the males in the 29 included couples (one couple was excluded because of difficulties in determining the ovulation date). Bars for standard deviation on the Y-axis are depicted against cycle day on the X-axis. No difference could be seen between day 4 and 14 in the multiple regression analysis (p < 0.971), and an estimation of similarity by type II error(β)-analysis using pairwise power calculation showed that the difference between these two points was less than 14.3% (β = 0.05).

Normalized salivary testosterone concentrations and registered confounders from the 29 included males (Figure 2) were analyzed by multiple regression analysis, revealing no difference in normalized testosterone concentrations between cycle day 4 (69.8 ± 14.9%) and 14 (68.4 ± 18.1%; p = 0.971). Similarity was estimated by type II error(β)-analysis using pairwise power calculation showing that the two points with β = 0.05 differed less than 14.3% (in terms of absolute normalized testosterone concentrations; Figure 3).

In the multiple analysis including all sampling days, none of the registered confounders (masturbation, intercourse, physical activity, alcohol consumption, illness or absence from partner overnight) were found to significantly affect the testosterone concentrations (p > 0.05).

### Population Characteristics

Age, average salivary concentrations, cycle length and frequencies of masturbation, intercourse, physical exercise and alcohol intake are presented in Table 1.

**Table 1:** Population characteristics

Males			
	Mean	SD	Range
Age [years]	28.9	5.6	22-46

Average salivary testosterone concentration [pg/mL]	178.9	44.1	108.1-276.6

Masturbation frequency [per 30-day month]	4.7	4.9	0-15.8

Intercourse frequency [per 30-day month]	6.1	4.2	0-13.78

Physical exercise frequency [per 30-day month]	5.9	5.9	0-20

Alcohol intake frequency [per 30-day month]	3.5	2.5	0-8.75

**Females**			

	Mean	SD	Range

Age [years]	26.9	5.6	20-45

Cycle length [days]	29	2.5	25-34

## Discussion

In contrast to the original hypothesis our results showed that the normalized testosterone concentrations on day 4 differed less than 14.3% (β = 0.05) from the concentrations on the day of ovulation. Even though individual males' testosterone concentrations fluctuated considerably, the accumulated result of all males rendered a flat testosterone-menstrual cycle curve (Figure 3). The high power of the study indicates that the results are strongly negative.

Even though human ovulation since long been considered concealed to the male partner [19], recent evidence has indicated that males may react to ovulatory stimuli. This includes studies that have demonstrated that males perceive near-ovulation odors as more pleasant than odors from women far from ovulation [20,21]. Further, the aforementioned pilot study in our lab indicated that the testosterone levels and acne of one male was associated to his wife's menstrual cycle (Figure 1). However, in contrast to these earlier results, the current study clearly indicates that the effect on males' testosterone levels by their female partners' ovulation in a real-life situation, on a group level, is at best minute. This does however not conclude that such an effect does not exist under any individual circumstances. It is possible that women differ in their capacity of emitting odorant ovulation cues or that men differ in the perception of these. Actually, 11 of the 29 included males in the current study had peaks (defined as the highest or second highest concentration) cycle day 13 to 15, however low concentrations of other males these days counterbalanced the mean concentrations. However, since such subgroup analyses were not planned in the original design, these observations should be assessed with caution.

The aforementioned article by Miller and Maner suggested that there was an effect on the male's testosterone levels by female ovulation odors, but the statistical evidence was actually weak. Males were allowed to sniff t-shirts worn by females near or far in time from ovulation, and even though the average testosterone levels seemed to decrease by both types of t-shirts, the decrease was smaller from sniffing the ovulation t-shirts [14]. In the second, most well-designed part of that study, the difference between ovulation t-shirts and non-ovulations t-shirts was merely that non-ovulation t-shirts were the only ones that significantly lowered the male testosterone concentrations. Strictly, no statistically significant difference was found between the ovulation t-shirts and non-ovulation t-shirts. This second part of the study was based on t-shirts from 11 women, and it could be speculated that inter-individual differences in these women's ability to emit ovulation odors may have weakened the observed phenomenon [14]. The clear effect in our own (based on one woman) pilot studies in contrast to the main study results further corroborates the notion that inter-individual differences may be an important confounder in studies of this phenomenon. Possibly the inter-individual variability is one of the components of partner compatibility. It has in several studies been demonstrated that body odor affects the choice of partner, and odor preference could theoretically also influence the impact of female ovulation on the male testosterone concentrations [22]. Another possible explanation to the discordant results is that the participating males may have been affected by body odors from other females than their partners, however, the present study design has not enabled us to study this factor.

It is also questionable whether our original hypothesis for the current study was correctly stated, or if e.g. other days than day 4 versus ovulation day should be the main comparison. However, the overall result as presented in Figure 3 is that the testosterone levels over the month are devoid of peaks, suggesting that the result would have been negative irrespective of the points chosen.

The design of the current study has attempted to control for and register all known influencing factors by a careful inclusion process, relatively large number of participants, pairwise design and control for confounders. Concerning the specific testosterone analysis, the well-reputed Salimetrics material was not only used for the biochemical analysis, but also for sampling, and to minimize pipetting errors a robot was used during the analysis, further adding to the reliability of the method. Increased precision in the assessment of daily testosterone concentrations could have been obtained by asking the participants to take several samples during an extended time-period every morning. This would however have made the study extremely demanding for the participants in exchange for a relatively small gain that most probably would not have affected the final conclusions.

Analyses regarding the effects of weather on testosterone concentrations, differences between the 11 couples having peaks days 13 to 15 and the other couples and correlation analysis between sexual intercourse frequency and masturbation frequency were also performed. However, since these aspects were not included in the original design, they are only briefly mentioned below, and not presented as parts of the Results. The weather parameters temperature, barometric pressure and air humidity were tested for correlation (Pearson's correlation) against male salivary testosterone concentrations, however no such relation was found (p = 0.14, 0.15 and 0.40 respectively). The 11 couples that potentially displayed testosterone peaks during ovulation neither differed in sexual frequency (p = 0.97), masturbation frequency (p = 0.63) nor age (p = 0.84) in comparison to the rest of the couples (Student's t-test). Concerning the relation between masturbation frequency and intercourse frequency, there was a significant negative correlation between the two parameters (r = -0.43, p = 0.021).

## Conclusions

Even though individual males' testosterone concentrations possibly can be affected by their respective female partner's menstrual cycle, this effect is very small or non-existing on a group level.

## Methods

### Participants

Young, healthy volunteers were recruited by posters and flyers on the university campuses in Linköping and Jönköping, Sweden. The posters and flyers specified that the participants must live together in a heterosexual relation, be 18-50 years of age and must not use hormonal contraceptives. After the participants contacted the research team, they were provided more detailed information about the study and were asked to answer an electronic inclusion/exclusion questionnaire. The information letter stated that the trial concerned how testosterone concentrations were affected by different social conditions, but the main hypothesis was not disclosed to the participants during the entire study.

The final selection of participants was made on the basis of the questionnaires, aiming to recruit a healthy and fertile group in which the hypothesized phenomenon was thought to be most likely to occur.

Inclusion criteria:

• Couple living together in a heterosexual relationship

• Premenopausal, and at least 18 years old

• Available during the study period

Exclusion criteria:

• Current or recent use of hormone-containing contraceptives

• Other fertile females living together with the participating couple

• Previous gonadectomy

• Transsexuality

• Consumption of drugs that could alter the sex hormone system

• Consumption of narcotics (anabolic steroids were specifically asked for in the questionnaire)

• Tobacco-smoking

• Disease that could affect the sex hormone system, social interaction or olfaction

• Planned absence from the partner during the trial

• Planned intensive physical exercise period (e.g. for an upcoming competition)

• Pregnancy or planned pregnancy during the time of the trial

• Oligomenorrhea

Of 73 couples initially signing up as volunteers, 48 adequately answered the questionnaires, of which 30 were included (Figure 2).

For the effort associated with participating in the study, each participant received the SEK equivalent of €150.

Before initiation of participant recruitment, the study protocol was approved by the Regional Ethical Review Board of Linköping University (2010/407-31).

### Sampling

Sampling for each participating couple started at a random time-point in the menstrual cycle, and proceeded five days longer than the duration of the female's longest menstrual cycle the previous year (from information provided in the questionnaire). Thus a female reporting a longest menstrual cycle of 30 days was asked to participate for 35 days, to make sure that at least one entire cycle was obtained. Saliva samples were taken at home the same time every morning by the participating males (Salimetrics oral swab, Item No. 5001.05 and Swab storage tube, Item No. 5001.02, Salimetrics Europe, Suffolk, UK). Any temporal deviations were registered in a protocol, but to avoid such errors all male participants were offered to receive a text message reminder on their cellular phones the same time every morning. The saliva samples were immediately put in a plastic bag and stored in the home freezer until the end of the sampling period. After the sampling period, all samples were collected by the research team, and stored in -20°C until analysis.

### Registration of Other Factors

In earlier studies, large quantities of alcohol [23], intense physical exercise [1], sexual activity [24,25] and illness [26] have been reported to influence testosterone levels. To control for these factors, all males received protocols in which they were instructed to record time of sexual activity (both masturbation and intercourse), physical activity, alcohol consumption, illness (defined as infection rendering fever or other illness warranting hospital visit) and absence from partner overnight. The females received protocols in which they were instructed to record first day of the menstrual cycle and day of ovulation. For detection of ovulation, hCG-tests for home use were provided (Wondfo hCG Urine Test, Cat. No. W1 - MII, Guangzhou Wondfo Biotech Co, Guangzhou, China). Right before the start of the trial, each couple met with one of the persons in the research team for detailed practical instructions and dialog.

### Testosterone Analysis

All samples were centrifuged for 15 minutes at 1500 g and analyzed by a salivary testosterone enzyme immunoassay (Item No. 1-2402, Salimetrics Europe, Suffolk, UK) using an analysis robot (Nexgen Four, Adaltis, Rome, Italy). This analysis kit has previously been thoroughly tested rendering intra- and inter-assay coefficients of variation of 3.3-6.7% and 5.1-9.6%, respectively [27].

The experimenter performing the analysis was blinded to the participants' menstrual cycle data, and since the samplings had started in random time-points in the cycle, the tests' analysis plate distribution was random.

### Statistics

#### Power calculation

The main comparison (prior to data collection) was selected to be between cycle day 14 (ovulation day) and day 4 of the cycle. These time-points were partially based on the aforementioned pilot study. In order to observe a 50% increase in testosterone levels between day 4 and 14, with a standard deviation of 40%, and a power of 0.99, a sample size of 25 pairs was needed (using unpaired power calculation and data from a previous study [12]).

#### Definition of cycle days

All data were adapted to a theoretical 28-day cycle, defined by the initiation of menses on day 1 and ovulation on day 14 (defined as the day after a positive ovulation test). The other days of the menstrual cycle were defined by the closest of these two definition points. Hence, in women who had cycles longer than 28 days, days that did not fit into the abovementioned algorithm were omitted.

#### Data analyses

Before analysis, each testosterone concentration was normalized by dividing by the highest concentration for every specific individual. The mean normalized salivary testosterone concentrations on days 4 and 14 (ovulation day) were compared using multiple regression, controlling for sexual activity, physical exercise, alcohol and illness on the day before the sampling. Samples that had been taken with more than 1 h delay were omitted (n = 5). If the difference between these points would not prove statistically significant, type II error(β)-analysis using pairwise power calculation was to be performed to estimate similarity.

Also, the impact of the variables sexual activity, physical exercise, alcohol, illness (the day prior to sampling) and more than 1 h delayed sampling on the normalized salivary testosterone concentrations were assessed by multiple regression in a second model including all sampling days.

All calculations were performed using Minitab (Minitab Inc., State College, PA, USA), and p-values < 0.05 were considered significant. Data are expressed as mean ± standard deviation throughout.

One participating female did not get a positive result on the ovulation test during the predicted period, but menses nonetheless started 14 days later. That couple was therefore excluded and omitted from all analyses.

## Competing interests

The authors declare that they have no competing interests.

## Authors' contributions

JOS handled all contacts with the participants recruited in Linköping, participated in the testosterone analysis and drafted the manuscript. EI participated in the testosterone analysis. ED handled all contacts with the participants recruited in Jönköping. All authors (JOS, EI, ED, AT and ET) participated in the design of the study, revision of the manuscript and approved the final version before submission.
